# Self-Harm and Suicide in Young People: Advancing Understanding and Intervention

**DOI:** 10.3390/healthcare14091151

**Published:** 2026-04-25

**Authors:** Jo Bell, Cathy Brennan

**Affiliations:** 1 School of Psychology and Social Work, University of Hull, Hull HU6 7RX, UK; 2 School of Medicine, University of Leeds, Leeds LS2 9NL, UK; c.a.brennan@leeds.ac.uk

Self-harm and suicide among young people represent one of the most pressing public health challenges facing contemporary societies. Over recent decades, rates of self-harm among adolescents and young adults have increased substantially in many countries, and suicide remains a leading cause of death among young people globally [1]. Self-harm is widely recognised as the strongest predictor of suicide, making early identification and intervention critical priorities for researchers, clinicians, and policymakers.

These behaviours are complex and cannot be understood through a single disciplinary lens [2]. Progress in the field increasingly depends on integrating psychological, social, cultural, and health-system perspectives. Research on self-harm and suicide among young people now spans qualitative studies of lived experience, theoretical models of suicidal behaviour, epidemiological investigations of risk and protective factors, and the development and evaluation of clinical interventions.

This Special Issue of *Healthcare*, *Health Risk Behaviours: Self-Injury and Suicide in Young People*, brings together contributions that reflect this multidimensional approach. The eight papers included in the collection examine the experiences of young people themselves, the psychological processes associated with self-harm and suicidal thinking, the influence of social and cultural environments, and emerging strategies for intervention and service delivery. Taken together, they offer new insights into the complexity of self-harm and suicide while also highlighting important gaps that remain in knowledge and practice.

Recent years have seen growing recognition that self-harm and suicide among young people arise from dynamic interactions between individual vulnerabilities, psychological processes, social environments, and structural inequalities. Traditional models focusing solely on psychiatric disorders are increasingly complemented by approaches that emphasise social determinants, developmental trajectories, and the lived experiences of young people and those who care for and support them [3,4].

One important development has been the expansion of qualitative research exploring how young people themselves understand and experience self-harm and suicidal thoughts. Such work highlights motivations that extend beyond clinical symptomatology, including emotional regulation, identity formation, interpersonal stress, and feelings of hopelessness or entrapment [5]. Another area of growing interest concerns the cognitive and motivational processes that may sustain self-harm behaviours. Emerging evidence suggests that self-harm may function as a coping strategy that provides short-term emotional relief or perceived reward, reinforcing the behaviour over time [6,7].

At the same time, there is increasing recognition of the broader social contexts shaping young people’s mental health. Digital environments, gender expectations, educational pressures, and sociopolitical conditions can all influence experiences of distress and help-seeking. Reflecting these developments, the field has also placed greater emphasis on the design and evaluation of early interventions, particularly in settings where young people first present in crisis, such as emergency departments, schools, and primary care [8,9,10].

The contributions to this Special Issue reflect these evolving priorities, offering insights that span individual psychological processes, lived experience, sociocultural context, and clinical response.

**Contributions of the Special Issue**

The eight papers in this Special Issue are best read not as isolated contributions, but as a connected body of work that advances the field across several levels of analysis: from the inner world of reward, motivation, and suicide rumination to the lived realities of young people in education and in families, to the wider social, cultural, and political conditions that shape risk, and finally to the question of what services can do, in practice, when young people present in crisis. In this sense, the Special Issue addresses self-harm and suicide in young people as both personal and structural phenomena, and as problems that demand simultaneous explanation, interpretation, and intervention.

Taken together, the eight papers build a picture of a field that is moving beyond single-cause explanations and towards a more layered understanding of self-injury and suicide in young people. The collection shows that these behaviours are shaped by disrupted motivational and cognitive processes, by relationships and institutional responses, by gendered and cultural expectations, by media ecologies, and by the larger sociopolitical conditions that determine whether young people experience their lives as viable and valued. Just as importantly, the Special Issue demonstrates that progress depends on methodological pluralism: qualitative work is needed to hear what distress feels like from the inside; quantitative work is needed to test pathways and moderators; theoretical work is needed to interpret rising risk in changing social contexts; and intervention research is needed to ensure that knowledge leads to better care.

**Future Research Priorities**

If this Special Issue shows anything clearly, it is that future research on self-harm in young people must become more integrative, more developmentally sensitive, and more structurally informed. The next phase of work in the field should not simply produce more studies of “risk factors” in the abstract. Rather, it should aim to explain how different forms of distress emerge, intensify, and are either mitigated or amplified across time, context, and systems of care.

First, there is a strong case for longitudinal and developmental research that can track the movement from distress to self-harm thoughts, from thoughts to acts, and from acts to repetition, recovery, or suicidal escalation. Several papers in this issue identify important pieces of the puzzle (reward processing, suicide rumination, hopelessness, stigma, and relational responses), but these are too often studied cross-sectionally or in isolation. Future studies should examine how these processes interact over time, and at what points intervention is most likely to alter trajectories. This is especially important in adolescence and emerging adulthood, when identities, relationships, and goals are changing rapidly and when self-harm may serve different functions at different developmental stages.

Second, future research should move decisively towards multi-level models of explanation. The field has made real progress in understanding individual-level correlates of self-harm and suicidality, but these alone are insufficient. The studies from Pakistan, Iraq, and the United States in particular make clear that structural adversity, gendered constraint, political climate, and social marginalisation are not secondary influences: they shape the very conditions under which despair becomes thinkable. A major priority, therefore, is to design research that can link subjective experience and clinical presentation with the wider determinants of mental health, including discrimination, educational exclusion, economic insecurity, conflict, and policy change. The challenge for the field is not merely to “add context” but to theorise and measure how context enters into risk and resilience.

Third, there is a pressing need for more culturally grounded and globally diverse research. Much of the dominant literature on self-harm and suicide among young people still reflects evidence generated in a relatively narrow range of countries and service settings. This Special Issue shows the value of broadening that evidence base, but it also exposes how much remains to be done. Future work should not assume that models, measures, or interventions developed in one context will automatically transfer to another. Instead, research should investigate how local meanings of distress, family obligation, honour, shame, help-seeking, and gender shape both risk and response. Comparative and collaborative international studies would be especially valuable, provided they avoid reducing cultural differences into simple variables.

Future studies should take more seriously the role of institutions and service systems, not only individuals. The research in this Special Issue shows in different ways that responses from universities, clinicians, emergency departments, and carers can shape whether a young person feels understood, dismissed, contained, or abandoned. Research priorities should therefore include how schools, colleges, universities, emergency services, and community mental health systems identify risk, respond after self-harm or suicide attempts, involve families, and manage continuity of care. Intervention development also needs to include implementation questions from the outset: what is feasible, acceptable, equitable, and sustainable in routine care, not just under ideal trial conditions?

There is also a need for more co-produced and young people-led research. A field concerned with young people’s survival and wellbeing should not routinely position them only as subjects (or participants) of study. Their involvement in framing research questions, interpreting findings, designing interventions, and shaping dissemination is essential if the work is to remain relevant and ethically grounded. This is particularly important in areas where adults’ assumptions may obscure young people’s realities, as the discourse analysis of media and self-harm usefully demonstrates. Co-production is not a methodological add-on; it is one route to producing knowledge that is less pathologising, more accurate, and more likely to improve support.

Finally, future research must engage more directly with the question raised most forcefully by Yen Li and colleagues: what makes life livable for young people [11]? The field has become adept at cataloguing risk, but prevention ultimately depends on more than identifying warning signs. It depends on understanding and strengthening the conditions that allow young people to experience belonging, dignity, safety, agency, and future possibility. That means studying protective environments as well as harmful ones; it means treating schools, communities, and policy contexts as sites of suicide prevention; and it means recognising that research on self-harm and suicide is inseparable from questions of justice. A stronger future agenda for the field will therefore be one that not only reduces harm but also helps build the social worlds in which young people can imagine staying alive.
